# Renormalized basal metabolic rate describes the human aging process and longevity

**DOI:** 10.1111/acel.12968

**Published:** 2019-06-11

**Authors:** Yasuhiro Kitazoe, Hirohisa Kishino, Kumpei Tanisawa, Keiko Udaka, Masashi Tanaka

**Affiliations:** ^1^ Center of Medical Information Science Kochi Medical School Nankoku Japan; ^2^ Graduate School of Agricultural and Life Sciences University of Tokyo Tokyo Japan; ^3^ Department of Physical Activity Research National Institutes of Biomedical Innovation, Health and Nutrition Tokyo Japan; ^4^ Faculty of Sport Sciences, Waseda University Tokorozawa Japan; ^5^ Department of Immunology Kochi Medical School Nankoku Japan; ^6^ Department for Health and Longevity Research National Institutes of Biomedical Innovation, Health and Nutrition Tokyo Japan

**Keywords:** allometric scaling law, human aging biomarker, mitochondrial number, mortality rate, renormalization of basal metabolic rate, survival curve

## Abstract

The question of why we age and finally die has been a central subject in the life, medical, and health sciences. Many aging theories have proposed biomarkers that are related to aging. However, they do not have sufficient power to predict the aging process and longevity. We here propose a new biomarker of human aging based on the mass‐specific basal metabolic rate (msBMR). It is well known by the Harris–Benedict equation that the msBMR declines with age but varies among individual persons. We tried to renormalize the msBMR by primarily incorporating the body mass index into this equation. The renormalized msBMR (RmsBMR) which was derived in one cohort of American men (*n* = 25,425) was identified as one of the best biomarkers of aging, because it could well reproduce the observed respective American, Italian, and Japanese data on the mortality rate and survival curve. A recently observed plateau of the mortality rate in centenarians corresponded to the lowest value (threshold) of the RmsBMR, which stands for the final stage of human life. A universal decline of the RmsBMR with age was associated with the mitochondrial number decay, which was caused by a slight fluctuation of the dynamic fusion/fission system. This decay form was observed by the measurement in mice. Finally, the present approach explained the reason why the BMR in mammals is regulated by the empirical algometric scaling law.

## 1 INTRODUCTION

Recent aging theories have proposed various causative biomarkers such as reactive oxygen species (Harman, 1956), calorie restriction (Faulks, Turner, Else, & Hulbert, 2006), telomere length (Aubert & Lansdorp, 2008), insulin signaling (Junnila, List, Berryman, Murrey, & Kopchick, 2013), mitochondrial (mt) DNA mutations (Linnane, Marzuki, Ozawa, & Tanaka, 1989; Trifunovic et al., 2004), fatty acid composition of membranes (Hulbert, Pamplona, Buffenstein, & Buttemer, 2007), and methylation (Hannum et al., 2013). To date, the validity of these biomarkers has been examined mainly by investigating their age dependency. However, they are not satisfactory for an accurate description of the aging process, and they seem to interact with each other in a complex way (Bratic & Larsson, 2013; Ernst, Haes, Cardoen, & Schoofs, 2014; Payne & Chinnery, 2015; Perez et al., 2009). Thus, it is essential to explain how these biomarkers can show that the survival curve and mortality rate are directly related to longevity. Indeed, the probability of survival drops markedly in individuals over the age of 80, and the mortality rate increases exponentially up to the age of 100 (Barbieri et al., 2015). In particular, a recent study reported that the mortality rate forms a plateau in centenarians and suggested that humans have the potential to live beyond the age of 120 (Barbi, Lagona, Marsili, Vaupel, & Wachter, 2018). However, this interesting result has to be confirmed by a robust theoretical analysis.

We here propose a new biomarker to describe the mortality rate and survival curve of the elderly. The basal metabolic rate (BMR) has long been known to decline with age, in line with the Harris–Benedict equation (HBE), which is useful for statistical analysis of a large amount of data (Harris & Benedict, 1918). The mass‐specific BMR (msBMR; BMR per unit mass) confers the standard normalization of BMR to decrease the variation based on the body weight of individual persons. However, the obtained msBMR still varies among them. We developed an approach in which a universal metabolic rate function of age was derived by renormalizing the msBMR. The first renormalization was attained by incorporating the body mass index (BMI) into the HBE. Interestingly, the variation of the msBMR was thus markedly decreased. We further performed a second renormalization to remove the remaining variation due to individual height by a little readjustment of the BMI. As a result, the renormalized msBMR (RmsBMR) revealed an exponential decline with only age (*T*) as a universal metabolic rate function irrespective of individual persons, *F*(*T*) = *F*(0) × e^−u^ *^T^* (*T* ≥ 16 years), with “u” as a decay constant.

First of all, we showed that the logistic model of the metabolic rate function *F*(*T*) accurately approximated the mortality rate of old Americans (>80 years of age; Barbieri et al., 2015) and also well reproduced the survival curve. Here, we recognized that the plateau of the mortality rate in centenarians (Barbi et al., 2018) was a critical signal for the lower limit (threshold) of the msBMR to maintain human life. The plateau effect prolonged the longevity especially without exceeding the age of 120.

The organ metabolic rates and weights of a reference male (Elia, 1992; Snyder et al., 1975) were useful to explain the reason why the RmsBMR provides the exponential decline with age. We found that renormalization of the msBMR corresponded to extraction of the core parts of organ weights from the body weights of individuals. This extraction was made by mainly adjusting the organ weights with low metabolic rates such as adipose tissue and skeletal muscle. We estimated the core organ weights and their msBMR of a number of American samples.

The RmsBMR is likely proportional to cellular metabolism and then to the mitochondrial number (mt density) within the standard cell. The exponential decay form of this density was shown to be a solution of the transport equation for the mitochondrial dynamical fusion/fission flow. This decay form was proven to be based on the Markov process, although the basic mechanism behind the occurrence of the mitochondrial dysfunction has remained unresolved (Payne & Chinnery, 2015; Seo et al., 2010; Westermann, 2012). The exponential decay of the mt density was supported by the measurements made in mice. Finally, the present approach also gave a theoretical basis to the empirical allometric scaling law in mammals.

## 2 RESULTS

### 2.1 Convergence of the msBMR and BMI variations with age

**Figure 1:**
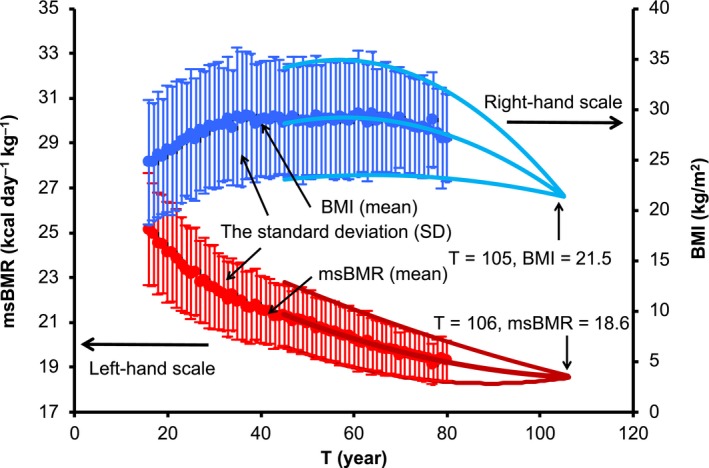
Age dependency of msBMR and BMI. The mass‐specific basal metabolic rate (msBMR) of 25,425 Americans was analyzed by using the Harris–Benedict equation (Harris & Benedict, 1918). The standard deviations (*SD*) of msBMR and BMI were calculated for the respective ages. The *SD* converged at almost the same ages of 105 and 106 with msBMR = 18.6 and BMI = 21.5. The solid curves are given as follow: msBMR(mean) + *SD* = −0.0058*T* ^2 ^+ 0.651*T* + 16.5, msBMR(mean) = −0.0035*T* ^2 ^+ 0.407T + 17.4, msBMR (mean) − *SD* = −0.0013*T* ^2^ + 0.162T + 18.3, BMI (mean) + *SD* = 0.0003*T* ^2^ − 0.111T + 27.2, BMI (mean) = 0.0006*T* ^2^ − 0.131*T* + 26.1, BMI (mean) − *SD* = 0.0008*T* ^2^ − 0.151*T* + 25.0

We analyzed a large number of American men (*n* = 25,425), aged 16–80 years and comprising those of 5 ethnic origins with various body shapes, by using data from NHANES (Centers for Disease Control & Prevention [CDC] National Center for Health Statistics [NCHS]). The analysis was initiated by determination of the age dependency of the msBMR in these Americans. Here, we applied the HBE (Harris & Benedict, 1918; Roza & Shizgal, 1984):

(1) $$\text{msBMR}=\left(88.362+13.397\times W+4.799\times H-5.677\times T\right)/W\left(\text{kcal}\;{\text{day}}^{-1}\;{\text{kg}}^{-1}\right),$$

in terms of body weight *W* (kg), height *H* (cm), and age *T* (years). We first calculated the mean value of the msBMR at each age and the standard deviation (*SD*) of it. The *SD* decreased with age and converged on “0” at *T* = 106 years with msBMR = 18.6 (kcal day^−1^ kg^−1^; Figure 1). Here, the 3 values of msBMR(mean), msBMR(mean) + *SD*, and msBMR(mean) − *SD* in the interval of *T* = 45 – 80 years were reproduced by second order polynomials, which were extrapolated toward larger *T*‐values. Interestingly, the *SD* of BMI showed a behavior similar to that of the msBMR and converged on “0” at *T* = 105 years with BMI = 21.5 kg/m^2^, which is typical for individuals with a healthy body shape. This result means that an individual with a better BMI would live longer.

### 2.2 Renormalization of the msBMR with a universal decline with age

**Figure 2:**
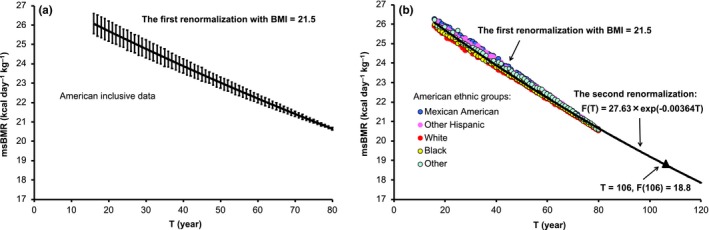
Renormalization of the msBMR. (a) The first renormalization with BMI = 21.5 markedly reduced the *SD* of msBMR, but the variations still remained. (b) The variation depended on the ethnic groups with different body shapes (solid circles). The second renormalization was attained by readjusting the BMI, and the result was given by the exponential function *F*(*T*) = 27.63 × exp(−0.00364T)

The msBMR (defined as BMR per unit mass) may represent the standard normalization of the BMR to decrease the variation of it in individuals. As was seen in Figure 1, however, the obtained msBMRs exhibited appreciable variations around the mean value. To remove the influence of these variations (primarily due to the body weight), we performed a two‐step renormalization of the msBMR. The first step involved applying the body weight given by BMI to the HBE. We used the body weight given by *W* = (*H*/100)^2^ × BMI, in which we assumed BMI = 21.5 kg/m^2^ (this value is equal to the convergent value of BMI at *T* = 105 (Figure 1) and is in the middle of the normal healthy range of 18–25). The results showed that the *SD* of the msBMR for the respective ages became very small despite the use of American samples with a variety of body shapes (Figure 2a). Moreover, the obtained msBMR was well reproduced by the following exponential decay function:

(2) $$F\left(T\right)=F\left(0\right)\times \exp\left(-uT\right),$$

with *F*(0) = 27.63 and *u* = 0.00364 (year^−1^).

Here, we observed a simple trend that the msBMR values of white and black Americans who were tall and those of other Americans who were short were generally plotted below and above *F*(*T*), respectively (Figure 2b). This trend means that the body weight given by BMI = 21.5 was still insufficient to describe the RmsBMR precisely, since a large body weight given by a tall height underestimated the msBMR and a small body weight given by a short height overestimated it. In this context, a second renormalization of msBMR was performed to readjust the body weight (also BMI) by equating msBMR given by the HBE to *F*(*T*) at each age. The resulting RmsBMR presented a universal exponential decay with age, irrespective of the individuals. We demonstrated the second renormalization effect by using typical samples with different body shapes (Section 4.2).

The RmsBMR may be written as the product of the number of cells per unit mass and the cell‐specific BMR (csBMR) in the standard cell, and the csBMR may be written as the product of the mt density and the mt output energy. Then, the csBMR is likely proportional to the mt density, since this density varies markedly among the organs according to their energy requirements (Table 1) and may also vary with age. In this sense, the mt density may be regarded as the primary biomarker of aging.

**Table 1:** Estimation of organ weights

	msBMR*_k_*(*r*)	*W*_0_ *_k_*(*r*)	*W_k_*(*r*)	*W*_0_ *_k_*(s3)	*W_k_*(s3)	*W*_0_ *_k_*(s4)	*W_k_*(s4)
Skeletal muscle	13.0	28.0	27.46	39.03	26.39	24.01	22.10
Liver	200.0	1.8	1.78	2.25	1.71	1.51	1.43
Brain	240.0	1.4	1.38	1.77	1.33	1.18	1.11
Heart	440.0	0.33	0.32	0.53	0.31	0.29	0.26
Kidneys	440.0	0.31	0.30	0.51	0.29	0.28	0.24
Adipose tissue	5.0	15.0	13.18	55.47	12.67	17.08	10.61
Residual	4.5	23.16	22.57	35.55	21.69	20.26	18.16
Total		70.0	67.0	135.10	64.38	64.60	53.92
Height		175.6		170.0		157.7	

Note

msBMR*_k_*(*r*) (kcal day^−1^ kg^−1^) and *W*_0_ *_k_*(kg), respectively, denote the original mass‐specific basal metabolic rate and body weight data of each organ in the 70‐kg reference male (Elia, 1992; Snyder et al., 1975). The renormalization procedure estimated the height *H*(r) = 175.6 (cm), the age *T*(*r*) = 32 (years), and the organ weights *W*_k_(*r*) of this male and also estimated the *W*_0k _(*i*) and *W*_k _(*i*) values of all other American samples. The results for 2 samples {s3 with *T* = 50 (years) and s4 with 80 (years)} are listed in this table. The renormalized organ weights *W*_k _(*i*) little depended on the body weights *W*_0 _(i), different from the original ones, *W*_0k_ (see Section 2.4 for details).

The renormalization procedure produced the universal metabolic rate function *F*(*T*) of the persons with a healthy body shape, although the data set (NHANES) included samples with a variety of body shapes within the age of 80. Indeed, the function *F*(*T*) expressed the exponentially decaying process with only 1 parameter, “*u*,” which was therefore related to the basic biomarker of mt density. This result strongly suggested that the function *F*(*T*) could be extrapolated to a wide range over the age of 80 in order to analyze the mortality rate. In fact, the mortality rate data could be very well reproduced in a simple form of the logistic function of *F*(*T*), as reported in the next section.

### 2.3 Mortality rate and survival curve in terms of the RmsBMR function *F*(*T*)

We first demonstrated the effectiveness of the present approach by elucidating the relationship between the metabolic rate function *F*(*T*) and the mortality rate. We here assumed that the death of humans over the age of 80 was caused by diseases deriving from the decline of *F*(*T*). Then, the mortality rate data could be well reproduced by the following equation:

(3) $$P\left(T\right)=\exp\left\{G(T)\right\}/\left[1+\exp\left\{G(T)\right\}\right].$$

Here, *G*(*T*) = *C* × {*F*(*T*_c_) − *F*(*T*)} with a constant *C*. *C* was determined so that *P*(*T*) may reproduce the observed mortality rate. *T*_c_ was defined as the age with the mortality rate of 0.5. The function *P*(*T*) in the form of the logistic function of *F*(*T*) can be interpreted as a Gomperz function modified for higher ages. This function is basically an exponentially increasing function of *T*, and it tends to be saturated in centenarians.

**Figure 3:**
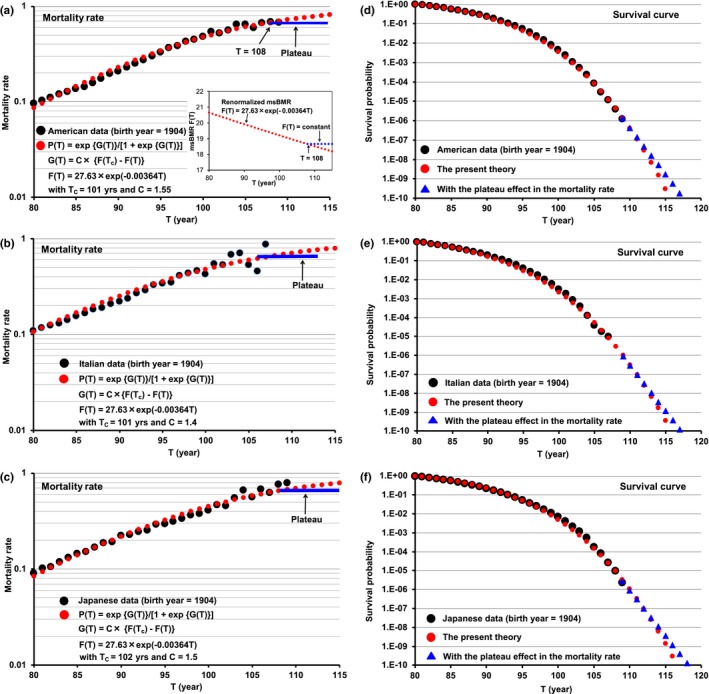
Mortality rate and survival curve expressed by using the universal metabolic rate function *F*(*T*). The black circles of Figures a, b, and c show the respective American, Italian, and Japanese data on the mortality rate (Barbi et al., 2018). These data were reproduced by using the logistic model function *P*(*T*) in Equation 3 (the red circles). The black circles of Figures D, E, and F on the survival curves show the respective American, Italian, and Japanese data. They were reproduced by transforming the function *P*(*T*) into the survival curve (the red circles). The blue lines of Figures a, b, and c reveal the formation of a plateau in centenarians over the age of 104 (Barbi et al., 2018). We put *P*(*T*) = 0.67 (*T* ≥ 108) for this plateau, which means that the metabolic rate function *F*(*T*) in Equation 2 is constant in centenarians (the insert in Figure 3a). The blue triangles of Figures d, e, and f represent the plateau effect reflected on the survival curve. This speculation strongly suggests that longevity would hardly exceed the age of 120

The black circles of Figure 3a–c show the observed respective American, Italian, and Japanese data on the mortality rate (Barbieri et al., 2015). The function *P*(*T*) well reproduced these data (the red circles), although it was derived in one cohort of the American data (Figure 2b). Here, the differences in the values of *C* and *T*_c_ in *P*(*T*) were very small (*C* = 1.55 and *T*_c_ = 101 for the American data, C = 1.4 and *T*_c_ = 101 for the Italian data, and *C* = 1.5 and *T*_c_ = 102 for the Japanese data). The black circles of Figure 3d–f show the American, Italian, and Japanese data, respectively, on the survival curve. The red circles in them were produced by transforming the function *P*(*T*) into the survival curve. The result was highly satisfactory despite a marked change in the survival curve.

It was recently pointed out that the Italian mortality rate data form a plateau in centenarians over the age of 104 (Barbi et al., 2018). We plotted this plateau on the U.S data by using the mortality rate (= 0.67) in *T* ≥ 108 (the blue line in Figure 3a). The blue line corresponded to putting *F*(*T*) = *F*(108) in the region of *T* ≥ 108 (the blue line in the insert of Figure 3a). The constant value of *F*(*T*) requires a balance between the decrease in msBMR based on the mt density decay and the increase in msBMR, which is caused by decreasing the body weight (mainly the adipose tissue). The state of this balance continues for several years, suggesting that the msBMR has a lower limit to maintain human life. The plateau prolonged the longevity (the triangles of Figure 3d–f), but the longevity was not likely to exceed the age of 120 because of a rapid decrease in the survival curve.

### 2.4 Organ analysis to support the renormalization of msBMR

We clarified the relationship between the change in the body weight given by the renormalization and that of the internal organ weights. The msBMR(*i*) of an individual sample (*i*) is expressed as follows:

(4) $$\text{msBMR}\left(i\right)=\sum_{k}W_{k}\left(i\right)\times {\text{msBMR}}_{k}\left(i\right)/W\left(i\right).$$

Here, *k* stands for the k‐th organ. *W*(*i*) denotes the renormalized body weight which is the sum of the renormalized organ weights *W_k_*(*i*), that is, *W*(*i*) = ∑*_k_W_k_*(*i*). The term msBMR(*i*) stands for the mean value of the organ metabolic rates msBMR*_k_*(*i*) with the weighting coefficient of *W_k_*(*i*).

The organ analysis was well performed by using a reference male (*r*) with the body weight *W*_0_(*r*), the organ weights *W*_0k_(*r*) [*W*_0_(*r*) = ∑*_k_W*_0_ *_k_*(*r*) = 70 (kg)], and the organ metabolic rates msBMR*_k_*(*r*) (Table 1; Elia, 1992; Snyder et al., 1975). At a given age *T*(*r*), a renormalized body weight *W*(r) was estimated by equating *F*{*T*(*r*)}of Equation 2 to msBMR with *W*(*r*) = {*H*(*r*)/100}^2^ × BMI in the HBE (1). We calculated a difference Δ*W_k_*(*r*) = *W*_0_ *_k_*(*r*) − *W_k_*(*r*) by distributing the reduction in the weight, *W*_0_(*r*) − *W*(*r*), so as to be proportional to msBMR*_k_*(*r*)^−^ *^q^* with *q* = 1.15. Then, we obtained *W_k_*(*r*) = *W*_0k_(*r*) – Δ*W_k_*(*r*). The difference ∆*W_k_*(*r*) was assigned so as to be large in adipose tissue, residual and skeletal muscle with low msBMR*_k_*(*r*) values (Table 1). By repeating this procedure, we determined *T*(*r*) so that msBMR(*r*) given by Equation 4 would be equal to *F*(*T_r_*). As a result, we obtained *T*(*r*) = 32, *W*(*r*) = ∑*_k_W_k_*(*r*) = 67.0, *H*(*r*) = 175.6, and msBMR(*r*) = 24.59 (Table 1).

The renormalized weights *W*(*r*) and *W_k_*(*r*) of the reference male made it possible to derive the renormalized organ weights *W_k_*(*i*) of the individual American samples (*i*) with the body weight *W*_0_(*i*), the height *H*(*i*), and the age *T*(*i*). We here rewrote Equation 2 as follows: *F*{*T*(*i*)} = *F*{*T*(*r*)} × exp[−*u*{*T*(i) − *T*(*r*)}], in which the origin of time was shifted at *T*(*r*) with *F*{*T*(*r*)} = msBMR(*r*) in Equation 4. Only the exponential term of *F*{*T*(*i*)} had the age dependency in the presence of various samples (*i*) with different ages, meaning that msBMR(*i*) of Equation 4 has to fulfill the condition *W_k_*(*i*)/*W*(*i*) = *W_k_*(*r*)/*W*(*r*), which gives msBMR(*r*) = msBMR(*i*) for any sample *i*. As a result, we obtained the organ weights *W_k_*(*i*) of the respective samples since the body weight *W*(*i*) was given by equating the function *F*{*T*(*i*)}to msBMR in Equation 1. Finally, we obtained the quantity Δ*W_k_*(*i*) by distributing the reduction in the weight, *W*_0_(*i*) − *W*(*i*), so as to be proportional to msBMR*_k_*(*r*)^–^ *^q^*. Then, we obtained the original organ weights, *W*_0k_(*i*) = *W*_k_(*i*) + Δ*W*_k_(i). The result of *W*_0k_(*i*) and *W*_k_(*i*) in 2 typical examples (s3 and s4) with different body shapes is demonstrated in Table 1. The organ weights strongly depended on the body weights of the individuals. The organ weights with smaller msBMR were much more variable compared with those with a higher msBMR, whereas the variation of the renormalized organ weights was markedly reduced. The BMI decrease with age in Figure 1 was associated with the body weight decrease in which the organ weights with a smaller msBMR decreased much with age.

**Figure 4:**
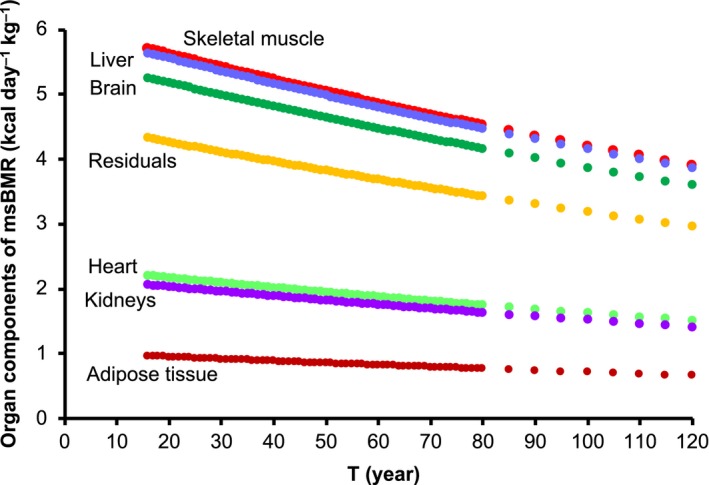
Exponential decay of organ compositions of the renormalized msBMR. We plotted the age dependences of the organ components of the renormalized msBMR

We plotted the exponential decay of the organ components of the renormalized msBMR in Figure 4. The ultimate death of a human corresponds to the situation in which all of the cells of the respective organs cannot play their specific roles because of the decline in cellular energy due to the loss of mitochondria. We here note that the decay constant “*u*” in Equation 2 stands for the mean value of the decay constant *u_k_* of individual organs. The organ analysis with different decay constants could be also done (Section 4.3).

### 2.5 Age dependency of the mt density in mice

**Figure 5:**
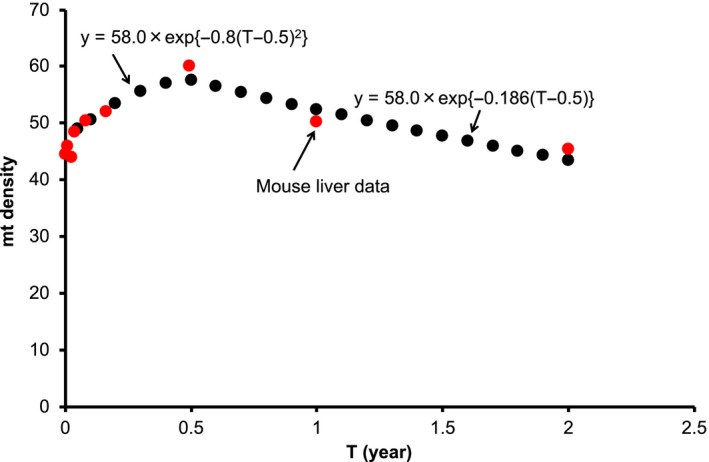
Age dependency of the mt density in mouse liver. There is precedent data demonstrating the age dependency of the mt density in mice (Nagata, 2006). We rewrote the metabolic rate function *F*(*T*) in Equation 2 as *F*(*T*) = *F*_0_ × exp{−30*u*(*T* − 0.5)} = 58.0 × exp{−0.186(*T* − 0.5)}, in which the maximum lifespan (120 years) of a human was scaled down to that (4 years) of a mouse. We used the decay constant *u* = 0.00624 for liver (Section 4.3). The function *y* = 58.0 × exp{−0.8(*T* − 0.5)^2^} was deduced so as to reproduce the data in the period of development up to adulthood (*T* ≤ 0.5 years)

The age‐dependency data on the mt density (liver) in mice was available (Nagata, 2006). To reproduce these data, we rewrote the metabolic rate function *F*(*T*) in Equation 2 as *F*(*T*) = *F*_0_ × exp{−30*u*(*T* − 0.5)}, in which the maximum lifespan (120 years) of a human was scaled down to that (4 years) of a mouse. We used the decay constant *u* = 0.00624 for liver (Section 4.3). The metabolic rate function *F*(*T*) somehow well reproduced the data in the aging period (*T* ≥ 0.5 years), as shown in Figure 5. The mt density appears to decrease with age in the aging period (Stocco & Hutson, 1978). More details of this density will be clarified in future work.

### 2.6 Theoretical support of the empirical allometric scaling law in mammals

**Figure 6:**
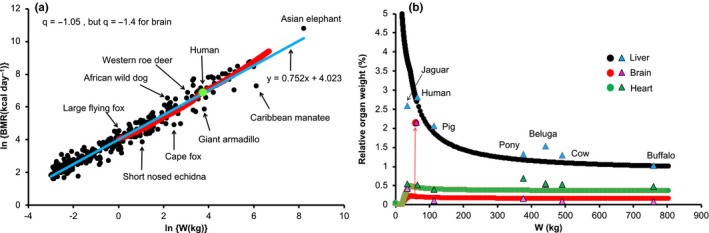
Application of the renormalization approach to the allometric scaling law in mammals. We here assumed that the relationship between BMR and the proper proportion of the renormalized organ weights in human holds for all mammals. Based on the optimized organ weights *W_k_*(*r*) of a reference male (Elia, 1992; Snyder et al., 1975), we analyzed how organ weights would behave when body weights were changed from 1 to 800 kg. We calculated the organ weights *W_k_* in each step of body weight change and then the metabolic rate BMR = ∑*_k_W_k_* × msBMR*_k_*. As a result, the obtained BMR was found to reproduce the observed data (de Magalhães & Costa, 2009)

The renormalization of the msBMR in humans provided a causal relationship between the body weight and the organ weights. We here propose that this relationship may be involved in a basic mechanism underlying the empirical allometric scaling law with a simple form (such as BMR ∝ *W* ^3/4^) in mammals. To examine the validity of this idea, we started with the renormalized organ weights *W_k_*(*r*) of the reference male (Table 1) and analyzed the behavior of organ weights in which the body weights of mammals were changed from 1 to 800 kg. We repeated the procedure for humans as follows: We considered the n‐th and (*n* + 1)‐th body weights, *W*(*n*) = ∑*_k_W_k_*(*n*) and *W*(*n* + 1) = ∑*_k_W_k_*(*n* + 1), in which *W*_k_(*n*) and *W*(*n* + 1) are given and *W*_k_(*n* + 1) is unknown. *W_k_*(*n* + 1) was determined under the condition that the difference ∆*W* = | *W_k_*(*n* + 1) − *W_k_*(*n*) | is proportional to msBMR*_k_*(*r*)^−^ *^q^*. Here, the constant *q* = 1.05 was used to reproduce the observed BMR data (de Magalhães & Costa, 2009). As the initial condition, we put *W*(1) = *W*(r) = 67 kg with *W_k_*(1) = *W_k_*(*r*). We used *W_k_*(1) = *W_k_*(*r*)/8.0 and *q* = 1.4 for only the brain, since the weight *W_k_*(*r*) of the human brain was too large to allow analysis of other mammals (the red arrow of figure 6b). The weight *W*(*n*) moved first from *W*(*r*) to 800 kg and next from *W*(*r*) to 1 kg (∆*W* = 0.5 kg was used as a small value). We obtained the organ weights *W_k_*(*n* + 1) in each step of the iteration and then the metabolic rate BMR(*n* + 1) = ∑*_k_W_k_*(*n* + 1) × msBMR*_k_*(*r*)/*W*(*n* + 1). As seen in Figure 6, the present approach well reproduced the observed data on both the BMR and the organ weights (de Magalhães & Costa, 2009; Crile & Quiring, 1940).

### 2.7 Mechanism of the exponential decay in the mt density

Mitochondrial morphology can change dramatically by shifting the balance between fusion and fission, which are essential for the quality control of mitochondria within the cell (Payne & Chinnery, 2015; Seo et al., 2010; Westermann, 2012). Fusion helps mitigate stress by mixing the contents of partially damaged mitochondria as a form of complementation. Fission is needed to create new mitochondria, enabling the removal of damaged mitochondria and facilitating apoptosis when there are high levels of cellular stress (Chan, 2006; Scheckhuber et al., 2007; Youle & van der Bliek, 2012).

We described the mt dynamic flow, which proceeds from biogenesis to mitophagy via fusion and fission within the standard cell, by using a transport equation. Interestingly, the solution to this equation for the mt number *N*_mt_(*T*) presented the same exponential form as that for the metabolic rate function *F*(*T*) in Figure 2b, *N*_mt_(*T*) = *N*_mt_(0) × exp(−*u* × *T*) with *u* = (P_13_ − P_11_)/(P_11_ × TOT) (Section 4.4). Here, P_13 _and P_11_ denote the 2 rates of mitophagy and biogenesis per unit time, respectively, and TOT is the mt turnover time. The exponential decline of the metabolic rate function *F*(*T*) with age strongly suggests that these 3 items are constants in normal aging (but they may change with age in mutator mice; Bratic & Larsson, 2013). Here, *N*_mt_(*T*) is the solution of the differential equation, d*N*_mt_(*T*)/d*T* = −*u* × *N*_mt_(*T*). This equation satisfies the Markov property in which the future states of the process depend only upon the present state; that is, given the present, the future does not depend on the past. In this way, the age dependency of *N*_mt_(*T*) can be explained as only a stochastic process.

## 3 DISCUSSION

### 3.1 Difference between the present biomarker and the previous ones

Here, we identified the RmsBMR as one of the best biomarkers to describe the mortality rate and related it to the exponential decay of the mt density. This mt density decay is derived from the fundamental mechanism of the mt dynamical system, different from the previous causative biomarkers which seem to interact with each other. For example, in the insulin signaling model, when the cellular metabolism begins to decline with age via a change in the level of a given hormone (Junnila et al., 2013), fission overpowers fusion (Chan, 2006; Kuhlbrandt, 2015; Scheckhuber et al., 2007). Then, the number of damaged mitochondria increases and the mt density decreases. Consequently, the cellular metabolism declines further. This vicious cycle between the cellular metabolism and mitochondrial dysfunction finally leads to death of the organism (van der Bliek, Shen, & Kawajiri, 2013). The mechanism of such a vicious cycle is also assumed in the oxidative damage theory (Bratic & Larsson, 2013; Harman, 1956; Seo et al., 2010). However, it is difficult to explain the mortality rate by the causative biomarkers with the vicious cycle, since this cycle accelerates the decrease in msBMR with age, compared with that of msBMR based on the Markov process without the causality.

### 3.2 Biological reason why the mitochondrial number decays with time

The mt number *N*_mt_(*T*) was found to decrease very slowly with age. Cellular energy is generated by a large number of mitochondria, which constitute about 10% of our body weight (Lane, 2005). Cells have to consume a large amount of energy (cost) even for producing the mitochondria themselves. Therefore, over the long history of evolution, these cells must have constructed a particularly efficient mt system that maximizes the cost–benefit ratio.

Mitophagy provides clear evidence that cells exert strong control over the quality of this system. However, even though this system is stable, effective, powerful, and robust for energy production, a stochastic error by which the equilibrium between biogenesis and mitophagy of mitochondria is slightly disturbed must occur. Indeed, when the metabolic rate function *F*(*T*) is proportional to the mt number, the decay constant “*u*” in this function provided a very small value, *u* = 0.00364/year. This means only a 0.36% decrease in the mt density during a single year, that is, 3.6 out of 1,000 mitochondria are lost in a cell during a single year, despite the occurrence of a number of biogenesis and mitophagy events. Such a small loss may be overlooked by the quality control of the cell, since stronger control would provide a less favorable cost–benefit ratio. Nonetheless, we should note that 1,000 mitochondria would become 685 mitochondria after a long aging period of 120 − 16 = 104 years. This inevitable constant decay of the mt density is a driving force of aging (Figure 2b; Section 4.4).

### 3.3 Signal of the final stage of human life

The RmsBMR in male persons with a healthy body shape steadily declines with age, becoming constant in centenarians over *T* = 108. This fact suggests that the RmsBMR cannot be further lowered to maintain life. The mt density still continues to decrease with age. However, it is here possible to keep the metabolic rate constant by decreasing BMI, that is, by mainly reducing the body weight. The plateau in the mortality rate suggests that the constant metabolic rate continues for several years, as the signal of the final stage of human life.

### 3.4 Biological meaning of the allometric scaling law

By starting with the optimal organ weights *W_k_*(*r*) and body weight *W*(*r*), and the metabolic rates msBMR*_k_*(*r*) of the reference male, we showed a simple way to estimate the organ weights *W_k_* of a mammal with the body weight W. Here, by distributing the difference |*W* − *W*(*r*)| so as to be proportional to msBMR_k _(*r*)^−^ *^q^* with *q* = 1.05, we determined *W_k_* and then BMR = ∑*_k_W_k_* × msBMR*_k_*(*r*). The quantity *W_k_* much depended on the body weight *W*. For example, the msBMR*_k_* of adipose tissue was one‐twentieth of that of the liver, and therefore, the weight of this tissue becomes markedly large as the body weight increases. As a result, the present approach reproduced the observed organ weights of mammals (Figure 6b) and explained why the BMR depends on the body mass, more clearly than the fractal geometry approach of allometric scaling (West, Brown, & Enquist, 1997).

### 3.5 Relationship between the present approach and modern aging theories

Modern biological aging theories consist of two main categories of programmed and damaged or error theories (Ernst et al., 2014; Kunlin, 2016; Payne & Chinnery, 2015). The programmed theories imply that the aging process follows a biological timetable (clock) regulated by changes in gene mutations, and the damage or error theories emphasize environmental assaults to living organisms. Indeed, in a previously programmed theory, a linkage of mitochondrial DNA mutations to aging was previously proposed (Trifunovic et al., 2004). However, it remains unclear whether such mutations play a fundamental role in the normal aging process (Bratic & Larsson, 2013; Sun, Youl, & Finkel, 2016). We recently reported that the maximum lifespan of mammals is related to the stability of the mitochondrial encoded‐membrane proteins (depending on the compositions of Ser, Thr, and Cys), that these compositions change in the evolutionary mammalian pathway over long periods, and that the substitutions (mutations) of these amino acids are minimal during the short lifespan (120 years) of human beings (Kitazoe, Hasegawa, Tanaka, Futami, & Futami, 2017).

The present approach belongs to the category of the programmed theories in the sense that the universal metabolic rate function *F*(*T*) constantly declines with age, on the basis of the exponential decay of the mitochondrial number. Some organs and tissues are likely to fall into a state of dysfunction in elderly individuals over the age of 80, since the supply of the cellular energy becomes gradually insufficient such that diseases manifesting due to the decline of this energy may cause the death of these humans. Here, dysfunctions are irregularly generated depending on the body condition of the individual persons. When all organs and tissues have no specific defects, longevity can exceed the age of 110, but cannot exceed the age of 120, because of the lower limit of the cellular energy needed to maintain human life.

### 3.6 Remaining problems in the present study

The renormalized metabolic rate function *F*(*T*) in Equation 2 described the universal age dependency of male persons with healthy body shape, as was seen in Figure 2b. When we follow the basal metabolic rates of the respective persons, some kinds of diseases will cause rapid and large deviations from this standard course at the organismal and organ levels. Classifying the age‐dependency patterns of these deviations will be useful for the diagnosis and treatment of diseases, since comparison of them with the function *F*(*T*) makes it possible to predict the forthcoming events of diseases.

## 4 METHODS

### 4.1 Primary procedure of the present aging analysis

We give an overview of the primary procedure in the present analysis:

- The analysis was initiated by determination of the age dependency of the msBMR by using the well‐known Harris–Benedict equation (Equation 1), and by using a large number of American men (*n* = 25,425) comprising those of 5 ethnic origins with various body shapes.
- Renormalization of the msBMR provided a universal metabolic rate function *F*(*T*) (equation 2) of age (Figure 2b).
- A simple form *P*(*T*) (equation 3) of the logistic function of *F*(*T*) excellently reproduced the American, Italian, and Japanese data on the mortality rate, without hardly changing the parameter value in *P*(*T*) (Figure 3a–c). By transforming the function *P*(*T*) into the survival curve, we obtained a good coincidence between the resultant survival curves and the observed survival curve data (Figure 3d–f).

### 4.2 Significance of the second renormalization of msBMR

We demonstrated the effect of the second renormalization by using 2 samples (s1 and s2) of *T* = 50 years. The first renormalization of the sample s1 with a tall height *H*(s1) = 187.7 (cm), a heavy weight *W*_0_(s1) = 114 (kg), and BMI(s1) = 32.4 (kg/m^2^) gave *W*(s1) = 76.6 with BMI(s1) = 21.5; and the second renormalization gave *W*_2_(s1) = 73.2 and BMI(s1) = 20.78. In contrast, the first renormalization of sample s2 with a short height *H*(s2) = 160.9, a light weight *W*_0_(s2) = 68.1, and BMI(s2) = 26.3 gave *W*(s2) = 56.3 with BMI(s2) = 21.5; and the second renormalization gave *W*(s2) = 59.9 and BMI(s2) = 23.14.

To summarize, the first renormalization provided msBMR_1_(s1) < *F*(50) < msBMR_1_(s2), since s1 and s2 had tall and short heights, respectively. In the second renormalization under the condition of msBMR(s1) = msBMR(s2) = *F*(50) = 23.0, the weight of s1 decreased from *W*(s1) = 76.6 to *W*_2_(s1) = 73.2 due to the tall height *H*(s1) = 187.7, whereas that of s2 increased from *W*(s2) = 56.3 to *W*_2_(s2) = 59.9 due to the short height *H*(s2) = 160.9. There is a physiological reason for the above result: A tall person generally possesses a larger amount of skeletal muscle to maintain body balance. This is associated with an increase in skeletal muscle weight with a low metabolic rate, thus lowering the msBMR (Figure 2b).

### 4.3 Organ analysis with different decay constants

When we use the renormalized body mass *W*(*i*) and organ weights *W_k_*(*i*) of any samples (i) belonging to *T* = *T*_0_, Equation 4 can be written as follows:

(5) $$\text{msBMR}\left(T_{0}\right)\times \exp\left\{-u\left(T-T_{0}\right)\right\}=\sum_{k}W_{k}\left(T_{0}\right)\times {\text{msBMR}}_{k}\left(T_{0}\right)\times \exp\left\{-u_{k}\left(T-T_{0}\right)\right\}/\sum_{k}W_{k}\left(T_{0}\right).$$

Here, *u* = 0.00364, *T*_0_ = 32, and *u_k_* denotes the decay constant of the k‐th organ. Since u (*T* − *T*_0_) << 1 and *u_k_* (*T* − *T*_0_) << 1, using exp{−*u_k_* (*T* − *T*_0_)} ≃ 1 − *u_k_* (*T* − *T*_0_), we obtain.

(6) $$\text{msBMR}\left(T_{0}\right)\times \left\{1-u\left(T-T_{0}\right)\right\}=\left[\sum_{k}W_{k}\left(T_{0}\right)\times {\text{msBMR}}_{k}\left(T_{0}\right)\times \left\{1-u_{k}\left(T-T_{0}\right)\right\}\right]/\sum_{k}W_{k}\left(T_{0}\right).$$

Then, we obtain the final expression for *u*,

(7) $$u=\sum_{k}{\text{BMR}}_{k}\left(T_{0}\right)\times u_{k}/\sum_{k}{\text{BMR}}_{k}\left(T_{0}\right),$$

with BMR*_k_* (*T*_0_) = *W_k_*(*T*_0_) × msBMR*_k_* (*T*_0_), and msBMR (*T*_0_) = ∑*_k_W_k_*(*T*_0_) × msBMR*_k_*(*T*_0_)/∑*_k_W_k_*(*T*_0_) = ∑*_k_* BMR*_k_*(*T*_0_)/∑*_k_W_k_* (*T*_0_).

By using the previous mouse data for TOT_k_ (Kadenbach, 1969; Menzies & Gold, 1971), we obtained *u*(skeletal muscle) = 0.00181, *u*(liver) = 0.00624, *u*(brain) = 0.00239, *u*(heart) = 0.00326, and *u*(kidneys) = 0.00532. We set *u*(adipose tissue) = *u*(residual) = 0.00364 as the mean value, since the TOT data were not available. We then estimated the age dependency of msBMR in the respective organs by using msBMR*_k_*(*T*) = msBMR*_k_*(*T*_0_) × exp{−*u_k_*(*T* − *T*_0_)} (Figure S1). Only the brain gave a different result from the case (Figure 4) with the mean u value. However, we noted above that the human brain is especially large compared with that of other mammals (Figure 6b).

### 4.4 Solution of the transport equation for mitochondrial fusion and fission process

There is a dynamic many‐particle system of mitochondria that continuously repeats fusion and fission within the standard cell. We describe the age dependency of this fusion and fission process with the help of the transport equation, which is given by the following differential equations:

(8) $${\left(N_{2}\right)}^{\prime }=-P_{21}\times N_{2}+P_{12}\times N_{1},$$

(9) $${\left(N_{1}\right)}^{\prime }=-\left(P_{12}+P_{13}-P_{11}\right)\times N_{1}+P_{21}\times N_{2}.$$

Here, *N*_1 _denotes the number of mitochondria that exist in the fission state; and *N*_2_, the number existing in the fusion state. *P_ij_* stands for the probability of mitochondrial transition from state *i* to state *j*. *P*_ij ×_ *N*_i_ gives the mitochondrial flow rate from state i to state j. *P*_11_ × *N*_1_ and *P*_13_ × *N*_1_ stand for the biogenesis and mitophagy rates of mitochondria, respectively. The first‐derivative Equations 8 and 9 can be rewritten in the second‐derivative form as:

(10) $${\left(N_{2}\right)}^{\prime \prime }+B\times {\left(N_{2}\right)}^{\prime }+C\times N_{2}=0,$$

with *B* = *P*_12 _+ *P*_21_ + *P*_13_ − *P*_11_ and C = *P*_21_ × (*P*_13_ − *P*_11_). As a result, we easily obtain the following solutions for *N*_1_ and *N*_2_:

(11) $$N_{1}\left(T\right)=N_{2}\left(0\right)\times \left\{\left(P_{21}+v\right)/P_{12}\right\}\times \exp\left(-uT\right),\;\text{and}\;N_{2}\left(T\right)=N_{2}\left(0\right)\times \exp\left(-uT\right)$$

with *u* = −{−*B* + (*B* ^2^ − 4*C*)}^1/2^/2. Here, since *P*_12_ + *P*_21_>> ▏*P*_11_ − *P*_13_▏ and *P*_12_ × *N*_1_ = *P*_21_ × *N*_2_, we have *u* ≃ *P*_21 _× (*P*_13_ − *P*_11_)/(*P*_12_ + *P*_21_) = *N*_1 _× (*P*_13_ − *P*_11_)/(*N*_2_ + *N*_1_) and *N*_1_(*T*) ≃ *N*_1_(0) × exp(−*uT*). As a result,

(12) $$N_{\text{mt}}\left(T\right)=N_{1}\left(T\right)+N_{2}\left(T\right)=N_{\text{mt}}\left(0\right)\times \exp\left(-uT\right).$$

Since the turnover time (TOT) is the period during which all of the mitochondria within the standard cell are replaced, we can put *N*_1_ × *P*_11_ × TOT = *N*_1 _+ *N*_2._ Then, we have

(13) $$u=N_{1}\times \left(P_{13}-P_{11}\right)/\left(N_{2}+N_{1}\right)=\left(P_{13}-P_{11}\right)/\left(P_{11}\times \text{TOT}\right).$$

As the ratio (*N*_1_/*N*_2_) increases, the decay constant “*u*” increases and TOT decreases.

## CONFLICT OF INTEREST

None declared.

## AUTHORS' CONTRIBUTIONS

Y.K. designed the study and analyzed the data. Y.K., H.K., K.T., K.U., and M.T. performed the research and wrote the paper.
