# Supplementary material for: Renormalized basal metabolic rate describes the human aging process and longevity
Source: Aging Cell. 2019 Jun 11;18(4):e12968. doi: 10.1111/acel.12968 (PMC6612648; doi:10.1111/acel.12968)
Supplement: Supplementary file 1 [file ACEL-18-e12968-s001.docx]

**Supporting Information**

**Figure S1. The age dependency of msBMR in the respective organs**
